# Advances in myopia prevention strategies for school-aged children: a comprehensive review

**DOI:** 10.3389/fpubh.2023.1226438

**Published:** 2023-08-15

**Authors:** Farheen Tariq, Rabia Mobeen, Xinhai Wang, Xiao Lin, Qingdong Bao, Jinhui Liu, Hua Gao

**Affiliations:** ^1^Shandong First Medical University & Shandong Academy of Medical Sciences, Jinan, China; ^2^Eye Hospital of Shandong First Medical University (Shandong Eye Hospital), Jinan, China; ^3^School of Ophthalmology, Shandong First Medical University, Jinan, China; ^4^School of Optometry and Vision Science, UNSW Sydney, Sydney, NSW, Australia; ^5^Shandong University of Traditional Chinese Medicine, Affiliated Eye Hospital of Shandong University of Traditional Chinese Medicine, Jinan, China; ^6^State Key Laboratory Cultivation Base, Shandong Provincial Key Laboratory of Ophthalmology, Eye Institute of Shandong First Medical University, Qingdao, China

**Keywords:** myopia prevention, school-aged children, outdoor activities, low-dose atropine, orthokeratology

## Abstract

Myopia has significantly risen in East and Southeast Asia, and the pathological outcomes of this condition, such as myopic maculopathy and optic neuropathy linked to high myopia, have emerged as leading causes of irreversible vision loss. Addressing this issue requires strategies to reduce myopia prevalence and prevent progression to high myopia. Encouraging outdoor activities for schoolchildren and reducing near-work and screen time can effectively prevent myopia development, offering a safe intervention that promotes healthier habits. Several clinical approaches can be employed to decelerate myopia progression, such as administering low-dose atropine eye drops (0.05%), utilizing orthokeratology lenses, implementing soft contact lenses equipped with myopia control features, and incorporating spectacle lenses with aspherical lenslets. When choosing an appropriate strategy, factors such as age, ethnicity, and the rate of myopia progression should be considered. However, some treatments may encounter obstacles such as adverse side effects, high costs, complex procedures, or limited effectiveness. Presently, low-dose atropine (0.05%), soft contact lenses with myopia control features, and orthokeratology lenses appear as promising options for managing myopia. The measures mentioned above are not necessarily mutually exclusive, and researchers are increasingly exploring their combined effects. By advocating for a personalized approach based on individual risk factors and the unique needs of each child, this review aims to contribute to the development of targeted and effective myopia prevention strategies, thereby minimizing the impact of myopia and its related complications among school-aged children in affected regions.

## 1. Introduction

Over the past three decades, there has been a noticeable upsurge in axial myopia among younger populations globally, with East and Southeast Asia experiencing a particularly significant surge (1). This increase in myopia prevalence has occurred alongside urbanization, an increased emphasis on education, and reduced time spent outside. The occurrence of myopia in secondary school students from East and Southeast Asia has escalated to encompass 80–90% of these populations (1), whereas many developed Western countries report rates of 20–40% (1–6). Conversely, young adults in less developed regions with underdeveloped education systems exhibit lower rates of myopia, typically less than 5–10% (7–10). According to projections, by the close of 2050, half of the world’s population will be impacted by myopia (11).

The etiology of myopia results from the interface between genetic and environmental factors. However, the sudden increase in prevalence of myopia over a short period contradicts the theory that genetic elements are the primary cause of myopia development. Instead, environmental factors such as educational and occupational behavioral demands, screen usage, reduced outdoor time, and inadequate exposure to sunlight are linked with an augmented risk of myopia (12). Individuals with low myopia (<−3 diopters [D]) are at significant risk. The escalating prevalence of myopia can be linked with an earlier onset of the condition, which consequently elevates the probability of advancing to high myopia. This pattern is especially problematic as high myopia, a more extreme variant of the condition, is correlated with a heightened susceptibility to severe ocular complications, including myopic maculopathy, retinal detachment, and glaucoma (13). Myopic maculopathy is the primary reason for blindness in nations such as Japan and China (14–16). Complications resulting from myopic maculopathy can profoundly affect an individual’s quality of life by causing permanent vision loss and disability.

To avoid the occurrence of severe myopia and its associated complications, as well as alleviate the financial impact caused by untreated or pathological myopia, it is necessary to implement appropriate measures. To decelerate myopia progression, multiple treatment options, including topical atropine, dual-focus contact lenses, multifocal soft contact lenses, overnight orthokeratology, and spectacle lenses, have been investigated. These approaches have exhibited a clinically meaningful reduction in advancing myopia (17–19). The optimal technique is chosen based on profession and geographical location (20). China has approved low-dose atropine (21) eye drops and orthokeratology lenses (22) for myopia prevention and treatment. The influence of behavioral changes, like augmented outdoor engagement and reduced screen exposure, on the pace of myopia progression remains uncertain (23).

While numerous studies have explored various methods for preventing and managing myopia (19), there is limited research that provides a comprehensive comparison of the efficacy of different environmental and optical interventions in preventing myopia in school-aged children. This review presents a comprehensive overview of myopia prevention strategies, highlighting the importance of integrating various approaches for optimal results. The goal is to contribute to developing targeted and personalized strategies that effectively mitigate the impact of myopia and its associated complications.

## 2. Methods used to conduct the review

This review was conducted by a thorough search of academic resources such as Google Scholar, and PubMed, specific search terms like ‘myopia,’ ‘myopia prevention,’ ‘school-aged children,’ ‘clinical trials,’ ‘atropine’, ‘contact lenses,’ ‘orthokeratology,’ and ‘myopia progression.’ Relevant articles were included in the study based on the following criteria: (1) availability of the full text; (2) written in the English language and (3) focused on myopia prevention strategies in school-aged children, myopia prevention and control, and randomized controlled trials prevention strategies, school-aged children (6–18 years), myopia prevention and control, randomized controlled trials and meta analysis. The article titles and abstracts were assessed for relevance, and those meeting these requirements were included in the analysis. Any discrepancies between the two authors were resolved through extensive discussions and consultation with additional specialists until a consensus was reached. Furthermore, a manual search was conducted by reviewing the reference lists of the included papers to ensure comprehensive coverage of relevant literature. After thorough consideration, a total of 104 articles were selected for inclusion in our review. It is important to note that animal studies, repetitive articles, articles published in languages other than English, and studies focusing on myopia prevention strategies in adolescents were excluded from this review.

## 3. Review of existing strategies

### 3.1. Environmental or lifestyle changes to control myopia progression

#### 3.1.1. Outdoor activities

Over the past few years, considerable research has investigated the association between myopia and outdoor activities in school-aged children (23–26). A randomized controlled trial (23) conducted in China assigned students to engage in 40 min of outdoor activities during school hours in addition to their regular curriculum. The intervention group showed a notable difference in the change of spherical equivalent refraction over 3 years (−1.42 D), in comparison to the control group (−1.59 D) (with a difference of 0.17 D [95% CI, 0.01–0.33 D]; *p* = 0.04). According to self-report questionnaires, nearly 10 to 14 h per week may eliminate additional myopia linked to increasing near-work or parental myopia (24). Efforts in Taiwan to reduce myopia, such as adjusting table height, improving room lighting, and instituting 39-min sessions of near work followed by 10 min break, did not significantly impact myopia prevalence rates (25). However, introducing an educational policy requiring a minimum of 80 min of daily outdoor time led to a substantial decrease in myopia incidence from 17 to 8% and a decline in the myopic shift from 0.38 to 0.25 diopters, particularly among children who had not yet developed myopia.

Likewise, a study (26) revealed that engaging in outdoor activities, particularly those that involved moderate to vigorous physical exercise, was correlated with a decreased risk of myopia in Chinese schoolchildren aged between 6 and 14 years. Conversely, a different study established that the safeguarding effect of outdoor exposure in preventing myopia seems to be more closely linked with the overall duration spent outsides than participation in athletic activities. This is because participating in indoor sports does not seem to be linked to a lowered likelihood of developing myopia (27). Outdoor activities, particularly those that involve extended outside periods time of 2–3 h per day for schoolchildren, are crucial in preventing myopia in school-aged children (28).

A recent prospective, cluster-randomized trial (29) aimed to evaluate the effectiveness of increasing outdoor hours per school day in halting myopia onset and progression. The research comprised a sample of over 6,000 students, aged 6–9 years, enrolled in primary schools in Shanghai, China, who were allocated to a control group or two test groups with additional outdoor time. The adjusted incidence of myopia was significantly lower in the test groups compared to the control group. Moreover, the test groups showed a notable reduction in myopic shift and axial elongation. The exposure duration and light intensity influenced the protective effect of outdoor time. A significant reduction in the incidence rate ratio by 15 to 24% was observed with a daily outdoor time of 120–150 min at 5000 lux/min or a cumulative outdoor light intensity of 600,000–750,000 lux. These findings highlight the importance of monitoring compliance with outdoor interventions to maximize the protective effect. Increasing outdoor time demonstrated a beneficial effect in reducing the risk of myopia onset and progression, particularly in nonmyopic children. Text message interventions were found to increase outdoor time and have a positive impact on reducing childhood myopia in a 1-year randomized trial (30). The SMS group showed significantly less axial elongation, a smaller myopic shift, and lower myopia prevalence compared to the control group. These benefits persisted at the 2- and 3-year follow-ups. The projected reductions associated with the SMS intervention align with China’s preventative objectives for myopia, with estimated decreases of 0.5 mm in axial length, 1.2 D in myopic shift, and 11.7% in myopia prevalence. Delaying the onset of myopia is expected to decrease the prevalence of high myopia.

Various theories and mechanisms have been posited to elucidate the underlying factors contributing to the protective effect of outdoor activities. These include augmented exposure to bright light, higher vitamin D levels, the influence of physical activity on myopia, decreased near work, and a reduction in accommodative demand (31). A review concluded that outdoor activities were related to a slower rate of axial elongation, which is a crucial factor in myopia development (32). Another study also suggested that exposure to bright outdoor light and peripheral defocus may protect the eye’s development (33). The idea that an augmented duration of outdoor exposure could potentially act as a shield against the onset of myopia was initially extrapolated from studies conducted on primates, which indicated that intensified illumination could trigger increased dopamine secretion from the retina (1). The increased dopamine secretion, in turn, might contribute to slowing down the axial elongation process, a key structural change associated with myopia. Therefore, it was inferred that higher light intensity could potentially prevent the occurrence of experimental myopia while not influencing other relevant factors (1).

There is a possibility that a prolonged period of outdoor pursuits, combined with physical exercise, could foster a healthier way of life for children and adolescents, potentially lowering the probability of obesity and other health complications (34). Integrating strategies to prevent myopia with measures to forestall excessive body weight gain could yield improved mental well-being and reduce depression, anxiety, and stress (27). Nevertheless, it is crucial to consider the possible risks associated with outdoor pursuits, such as heightened skin cancers, sun exposure, and contact with harmful environmental substances. However, recent evidence suggests that visible light, rather than UV radiation, may be the causal factor in preventing myopia. Therefore, interventions to prevent myopia should be compatible with measures to avoid UV exposure (25).

It is worth noting that using red light (with a wavelength of 650 nm) through a desktop light therapy device at home has recently demonstrated effectiveness in controlling myopia. During the 12-month follow-up examination, the group receiving red light treatment exhibited a remarkable 70% decline in myopia progression. Additionally, 32% of participants in this group experienced a decrease in axial elongation of at least 0.05 mm. These findings were reported in reference (35). However, further investigations involving double-masking and placebo-controlled groups are necessary to fully comprehend this approach’s long-term efficacy and safety, as well as to explore potential rebound effects and determine optimal treatment strategies. These further investigations will also help us comprehend the fundamental mechanisms at play.

#### 3.1.2. Reduced near-work and screen time

Behavioral approaches, which typically aim to decrease visual activity or near work that requires a significant amount of accommodation, are gaining popularity. Research indicates that engaging in near-work activities has been categorized with a higher likelihood of developing and worsening myopia (36–38). In the last 20 years, electronic gadgets like computers and smartphones have become customary daily, with many countries integrating them into their educational systems. Thus, it is reasonable to relate higher usage of digital devices to more time spent indoors and engaging in activities that demand near work, which could increase the probability of myopia onset and development in youngsters (39). Lu and colleagues (40) conducted a cross-sectional study that revealed a correlation between longer screen time and a higher incidence of myopia among Chinese children. The study also indicated that minimizing screen time could be an effective approach to prevent myopia.

A substantial cohort study called the Generation R study (41), which included 5,074 children from Rotterdam, found a connection between myopia and augmented hours of computer usage among 9-year-olds (OR: 1.005 and 95% CI: 1.001–1.009). The research also showed that the likelihood of getting myopia at 9 years of age rose with the expansion of near-work assignments, such as reading, computer usage, and reading distance (OR 1.072 and a 95%CI: 1.047–1.098) (41). Furthermore, a recent study has demonstrated a significant positive correlation between smartphone usage and an increased risk of developing myopia. The analysis revealed a connection between exposure to screens, increased myopic spherical equivalent, longer axial length, and prevalent and incident myopia. The study demonstrated that using smart devices alone (OR 1.26 and 95% CI: 1.00–1.60) or when combined with computer usage (OR 1.77 and 95% CI: 1.28–2.45) was significantly linked with myopia (39). A widely accepted clinical suggestion, likely derived from the research above, is the 30-rule: maintaining a working distance of over 30 cm and taking 30-s breaks for every 30 min spent on close-up tasks. In China, products like the Clouclip are promoted as protective measures against myopia-causing habits. The Clouclip notifies children (through vibrations) and parents (*via* an app), when a kid’s watching patterns are considered detrimental (viewing distance <30 cm and > 5 s; viewing distance <60 cm for >45 min) (42).

Several studies have explained the connection between screen usage and the emergence of myopia. One explanation concerns tasks requiring close-up vision, which includes the impacts of excessive accommodative convergence and peripheral defocus on the elongation of the axial length of the eye (43). Another factor is the smaller screens and font sizes on smart devices that necessitate closer viewing distances, resulting in increased demands on accommodation and vergence compared to print materials (44). Additionally, utilizing screens indoors limits exposure to outside surroundings’ protective features, such as higher luminance and more stable dioptric space, which may prevent emmetropization from occurring (45). Reduced retinal dopaminergic neurotransmission brought on by reduced sunlight, a crucial mechanism in maintaining proper refractive growth, may be partially blamed for this disturbance (46).

The COVID-19 pandemic has caused people to rely more on digital devices due to lockdown measures, which has led to concerns about the potential increased risk of myopia (47). In contrast to the years 2015–2019, a noticeable increase in myopia progression (approximately −0.3D) was observed during the 2020 school-based photo examinations, particularly among younger children aged 6 (−0.32 D), 7 (−0.28 D), and 8 (−0.29 D). The prevalence of myopia detected in the 2020 photo exams surpassed the highest recorded levels between 2015 and 2019 for children aged 6 (21.5% compared to 5.7%), 7 (26.2% compared to 16.2%), and 8 (37.2% compared to 27.7%) (48). To assess the prevalence of myopia and its related factors before, during, and after COVID-19 restrictions, a cross-sectional study was undertaken in Hong Kong. The study involved children aged 6 to 8 years and observed that the prevalence of myopia remained steady before the pandemic but significantly increased (1.5 folds) during and after the easing of restrictions. The study also found a decrease in outdoor time during the pandemic, which did not fully recover afterward. Younger age, male sex, lower family income, and parental myopia were identified as factors associated with higher myopia prevalence. During the pandemic, children from lower-income families exhibited greater near-work time and screen time. These findings underscore the importance of targeted myopia control measures, particularly for younger children and those from low-income backgrounds, in addressing the impact of the COVID-19 pandemic on myopia development (49).

Wong and colleagues (50) reviewed studies examining the association between using PCs, tablets, or smartphones and myopia. While the evidence is not definitive, most studies suggest that spending more time on digital screens is associated with a higher risk of myopia. The authors cautioned that the pandemic could exacerbate myopia by increasing exposure to digital devices and that long-term adverse effects may be associated with their use (50). A study conducted in China demonstrated the efficiency of a school-based intervention program in reducing children’s screen time and slowing the progression of myopia. The program included educational components to raise awareness about the detrimental effects of excessive screen time and the significance of taking regular breaks. The findings of the study supported the hypothesis that such interventions can be beneficial in mitigating the onset and advancement of myopia in children (51). Schools have begun restricting screen time in mainland China to curb myopia (52).

### 3.2. Optical or pharmacological interventions to control myopia progression

#### 3.2.1. Optical interventions

Vision is the primary determinant of ocular development. When a negative lens artificially displaces the image plane posterior to the retina, it induces hyperopic defocus. This stimulus triggers ocular growth, ultimately leading to the development of relative myopia. Conversely, when a positive lens shifts the image plane anterior to the retina, it generates myopic defocus. This condition inhibits ocular growth and promotes the development of relative hyperopia. Based on this underlying principle, various optical approaches have been devised in recent years to reduce the advancement of myopia. Initially, research on optical treatments for myopia progression primarily investigated the impact of under-correcting the myopic refractive error and utilizing conservative bifocal spectacles (53). Nonetheless, research has yet to conclusively show any benefits related to overcorrecting or under-correcting myopic refractive errors or employing monovision, as indicated in recent Cochrane and systematic review publications (18, 54).

Similarly, studies on optical under-correction of myopia have yielded inconclusive results, indicating that it either had no effect or seemed to accelerate myopia progression (55). Research has indicated that having a peripheral myopic defocus can be beneficial, as demonstrated in studies that compared kids wearing progressive addition lenses with single-vision lenses (56). “Bifocal spectacles have shown the potential to decelerate myopia advancement in children who experience a yearly progression rate of at least 0.50 D over 3 years. The findings indicate that prismatic bifocals may be particularly beneficial for children with minimal accommodative lags, offering an effective management strategy for myopia (56). Three optical choices are available: Defocus Incorporated Multiple Segments (DIMS) eyeglasses (57, 58) concentric zone dual-focus soft contact lenses that correct and induce myopic defocus (59–61), and Orthokeratology (OK) contact lenses (22, 62).

##### 3.2.1.1. Dual or multifocal focus spectacles lens

In clinical research comparing children wearing progressive addition lenses to those wearing single-vision lenses, peripheral myopic defocus is advantageous. Lam et al. (58) recently created the DIMS lens. In contrast to prior lens designs, the peripheral myopic defocus zone of the DIMS lens features a revolutionary design of honeycomb multizone with a + 3.50 D myopic defocus sector and a clear sector with central power. In a randomized, controlled clinical experiment conducted by these researchers, the DIMS lens decreased myopia development by 59% and repressed axial development by 60%, linked to the typical single vision (SV) lens50 (58). Given that spectacle-based myopia therapies with positive dioptric power may have minimal effects on peripheral vision, evaluating this aspect is crucial (57). In a three-year follow-up study, children who continued to use DIMS lenses or transitioned from single vision (SV) lenses to DIMS lenses for 1 year after a two-year myopia control experiment were evaluated for myopia progression. A total of 128 children participated in the study. The findings indicated no statistically significant changes in cycloplegic SER and AL in the DIMS group throughout the 3 years. In the Control-to-DIMS group, the changes in SER and AL during the third year were significantly lower compared to the first and second years. The two study groups demonstrated significantly fewer variations in SER and AL than the historical control groups. These findings highlight the sustained myopia control effect in children who used DIMS lenses for 2 years and the effectiveness of swaping from SV to DIMS lenses in controlling myopia progression.

Similarly, another study (63) evaluated a lens design with concentric rings of aspheric lenslets for myopic defocus. In a trial with three different treatment options, children aged 8 to 13 were arbitrarily assigned to use SVLs, lenses with highly aspherical lenslets, or mildly aspherical lenslets. The study found that eyeglasses with aspherical lenslets were more efficient in reducing the advancement of myopia and axial growth compared to SVLs. Additionally, the research showed that the effectiveness of myopia treatment improved with increasing lenslet asphericity. Although there is a link between dual-focus spectacle wear and bicycle accidents in children, there is no corresponding association between the incidence of such crashes and myopia or habitual visual acuity (64). According to the researchers, the rise in risk is linked to a decrease in the rider’s peripheral visual field, which lowers their ability to detect approaching vehicles and obstacles on the road. Improving myopia and eliminating blurry vision have advantages of their own. In contrast, no proof exists that children are exposed to the same hazards. Adults who use progressive addition or bifocal lenses have a two-fold increased risk of falling compared to those who do not wear multifocal lenses (65). Still, it is uncertain whether the same risk applies to children, as they rarely use such lenses.

##### 3.2.1.2. Dual or multi-focus soft contact lenses

In recent years, there has been a growing interest in utilizing soft contact lenses as a potential strategy to impede myopia progression. Soft multifocal contact lenses have been developed to optimize visual acuity and decelerate ocular growth by directing some incoming light onto the retina and some in front of it. Several randomized controlled trials have been executed on the efficacy of soft multifocal contact lenses. The findings demonstrate that these lenses lead to an average decrease in myopia development by 36.4% and axial elongation by 37.9% (54, 66–68).

Significantly, the utilization of the MiSight soft contact lens, which incorporates a clear middle distance zone and concentric rings with comparative plus power, has been shown to effectively reduce spherical equivalent refractive error over 3 years among individuals who use it. Compared to the control group, which experienced a reduction of 1.24 ± 0.61 D, the study group demonstrated a significantly lesser reduction of 0.51 ± 0.64 D, representing a 59% reduction. Moreover, there was a 52% reduction in the mean change in axial length, with the study group showing a change of 0.30 ± 0.27 mm compared to the control group’s change of 0.62 ± 0.30 mm. These compelling findings led to the approval of the MiSight multifocal contact lens by the “United States Food and Drug Administration” as a daily and one-time treatment to slow down the progression of myopia in children effectively. The approval was based on a rigorous three-year clinical trial conducted across multiple centers (66).

To evaluate the efficiency of soft multifocal contact lenses in preventing or halting myopia progression, the BLINK trial (68) included 292 participants with a mean age of 10.3 ± 1.2 years. Subjects were randomly assigned to wear either single-vision contact lenses or contact lenses featuring central myopia correction, along with either a high add (+2.50 diopters) or medium add (+1.50 diopters) power in the peripheral concentric zone. After 3 years, the researchers found that the group with the highest add power exhibited the most substantial difference in controlling myopia progression rate than the other two groups. The results demonstrate that soft multifocal contact lenses can halt myopia progression and eye growth. However, there is still uncertainty regarding the perfect distribution of refractive power to attain the most effective halting of myopia progression without negatively impacting visual function (68). A recent study (61) compared the vision-related quality of life (VRQoL) between Defocus Incorporated Soft Contact (DISC) lenses and SVL in Chinese children with myopia. Chinese individuals aged 7 to 12 years wore either DISC lenses or SVL for 6 to 18 months. The participants completed ocular examinations and a questionnaire (Pediatric Refractive Error Profile) to evaluate their VRQoL. The results showed children wearing DISC lenses had significantly higher scores in vision, peer perception appearance, activities, and overall VRQoL than those wearing single-vision spectacles. Older participants experienced more significant improvements in vision-related aspects such as symptoms, handling, appearance, and overall VRQoL. There was a significant association between treatment and age in relation to the activities scale.

The potential risks related to using soft contact lenses have been extensively reported. Non-infectious inflammation can impact the conjunctival and periorbital tissues. A retrospective observational investigation was conducted to identify the obstacles hindering the adoption of soft contact lenses among children, adolescents, and young adults (69). The study involved 3,549 individuals who experienced 187 cases of corneal infiltrates over a period of 14,305 visits and 4,663 years of soft contact lens usage. Forty-one peripheral ulcers in contact lens wearers, as well as 14 cases of acute red eyes with infiltrates and 13 cases of acute red eyes without infiltrates, were identified. Soft contact lenses were worn for an average of 20 months by the 1,054 patients under 18 years old. The study found a non-linear association between age and the possibility of corneal infiltrative events, with the highest risk being observed between the ages of 15 and 25. The data analysis from nine studies revealed 14 corneal infiltrative occurrences, resulting in an incidence rate of 78 per 10,000 patient-years (95% uncertainty rate, 44–127 per 10,000 patient-years). According to the research, none of the studies included in the analysis described any instances of microbial keratitis, with a 95% uncertainty interval of 0 to 21 per 10,000 patient years. Similarly, a retrospective study examining over 800PYs of soft contact lens usage in children uncover no microbial keratitis (70). However, in the latest clinical trial involving almost 900PYs of soft contact lens use among children, one incidence of “presumed” microbial keratitis was reported (68).

In short, the chances of corneal infiltrative infection and microbial keratitis in kids younger than 12 years old, who are more prone to high myopia progression, do not appear to be greater than that in adults and might even be lesser (71). The age range with the highest rate of complications is between 18 to 25 years, indicating the impact of behavioral and lifestyle factors (72). Parents are usually more involved in the lens care of 8- to 12-year-olds, and it is plausible that young children who wear contact lenses are more responsible for their use. However, if a more significant proportion of children begin to wear contact lenses, the current low incidence rate of complications may potentially increase.

##### 3.2.1.3. Orthokeratology

In orthokeratology, the cornea’s curvature is temporarily altered by wearing custom-designed, gas-permeable contact lenses with a reverse geometry design. The lenses are worn overnight, primarily to decrease daytime nearsightedness by causing the central cornea to become flatter. This effect is believed to occur due to the rearrangement of the corneal epithelium’s multiple layers, leading to a thinner central corneal epithelium. In recent times, numerous clinical trials have exhibited that wearing orthokeratology lenses overnight can effectively delay the advancement of myopia in children (73). Additional research, primarily focused on children and teenagers, has demonstrated that orthokeratology may also contribute to a reduction in the axial length of the myopic eye. This effect could be attributed to a decline in relative peripheral hyperopia due to the steepening of the mid-peripheral corneal zone (74, 75).

In two distinct randomized controlled studies, the Retardation of Myopia in Orthokeratology (ROMIO) study by Cho et al. (74), and the HM-PRO trial by Charm and Cho (76), a reduction of axial elongation ranging from 43 to 63% was detected. In the control group, the proportion of younger children (aged 7–8 years) with fast myopic progression (>1.00 D per year) was 65%, while for older children (aged 9–10 years) it was 13%. In comparison, the ortho-k group had a lower proportion, with 20% of younger children and 9% of older children experiencing fast myopic progression. However, a limitation of the ROMIO study was the 27% dropout rate in the intervention group. Throughout the two-year trial, which included children with a refractive error of at least −5.75 diopters, the treatment group exhibited a median myopia progression of 0.13 diopters. In contrast, the glasses-wearing control group showed a significantly higher median progression of 1.00 diopters (74, 77). This study had a high fallout percentage of roughly 50%. A recent meta-analysis reported that orthokeratology (OK) has a moderately beneficial effect (76).

Similarly, in another recent meta-analysis of 13 randomized clinical studies (22), Orthokeratology (Ortho-K) has demonstrated the ability to reduce axial length elongation by approximately 50% over a two-year research duration. The average difference in axial length change between Ortho-K patients and control patients was about 0.3 mm and 0.6 mm, respectively. This resulted in an average difference in refractive error of around 0.5 diopters (D). Ortho-K is an efficient method for retarding myopia progression in pediatric and adolescent populations, as it reshapes the cornea and provides temporary refractive correction, especially when initiated between the ages of 6 to 8 years (22). During the three-year follow-up period of the CLAMP Study (78), myopic development was substantially lower in RGP lens wearers than in single-vision lens wearers. Additionally, those who used single-vision lenses experienced greater corneal curvature steepening than those who wore RGP lenses; however, there was no significant difference in ocular growth between the two groups. However, in a randomized trial (79) conducted in Singapore over 2 years among pre-adolescent school students, no substantial evidence was found that RGP lenses could reduce myopia progression. The study included children wearing contact lenses for 12 or more hours daily. Still, although there was a slight difference of 0.2 diopters in the rate of progression between the contact lens and spectacle groups, the difference was not statistically significant. Nonetheless, it is worth noting that a substantial number of children in the study could not tolerate or maintain prolonged use of RGP contact lenses throughout the study.

Another prospective, randomized, contralateral-eye crossover study (73) investigated the impact of nightly orthokeratology (OK) contact lens usage on axial elongation in children of East Asian origin with progressive myopia. The study lasted 1 year, during which participants were fitted with nightly ortho-K lenses for one eye and conservative rigid gas-permeable glasses for daytime wear on the other eye. Both sets of lenses were worn simultaneously. The study findings indicated that wearing OK lenses overnight had a more pronounced repressive effect on ocular growth in children with progressive myopia than regular GP lenses during the daytime. Despite this positive result, orthokeratology has yet to gain widespread acceptance for several reasons. These include the potential requirement for additional training for doctors to install ortho-K lenses, discomfort while wearing the lenses overnight, the high cost of the lenses themselves, and the possibility of developing an infection in the cornea known as infective keratitis (80). Jakobsen et al. (81) conducted a randomized study involving 60 Danish children aged 6 to 12 years assigned to ortho-K lenses or single-vision spectacles (SVS) and monitored them for 18 months. The ortho-k lenses group showed a statistically significant reduction of 0.24 mm (95% CI 0.12–0.36) in axial length (AL) compared to the SVS group, with a 95% CI of 0.12–0.36. In contrast to the SVS group, no fast progressors (>0.75 D/year) were observed in the ortho-K lenses group during the follow-up period, while 22% of the SVS group did. Additionally, no significant adverse events that required treatment or affected vision were reported.

The Variation of Orthokeratology Lens Treatment Zone (VOLTZ) Study (82) aimed to explore the effect of ortho-k lenses with different back optic zone diameters (BOZD) on myopia control over 1 year. Children aged 6 to <11 years with myopia ranging from −4.00 D to −0.75 D were randomly assigned to wear either 6 mm (6-MM group) or 5 mm (5-MM group) BOZD ortho-k lenses. The study found that both lens types demonstrated similar clinical performance and did not negatively impact ocular integrity. However, the 5-mm BOZD lens resulted in a smaller treatment zone (TZ) size and slower axial elongation than the 6-mm BOZD lens. These results suggest that ortho-k lenses with a smaller BOZD may effectively control myopia progression in children. Chen et al. (75) researched the efficacy of toric Ortho-K lenses in slowing myopia development in children with moderate-to-high astigmatism. Based on their prior experience with spherical Ortho-K lenses, they sought to determine if toric Ortho-K lenses could yield better results. After monitoring for 24 months, the researchers found that children who used toric Ortho-K lenses had a 52% reduction in axial length elongation compared to the control group, which wore single-vision glasses.

Considering the initial investment and ongoing expenses associated with this approach is essential. Compared to traditional eyeglasses or contact lenses, Ortho-K typically requires a higher initial investment, which may make it less accessible for some families. Alongside the upfront cost of the lenses, patients must also account for professional fees, lens replacement, and maintenance supplies that can accumulate over time. In the US, expenses associated with contact lenses are generally not included in standard insurance policies, and the prices for Ortho-K exceed those of conventional contact lenses or eyeglasses (presently, around $1,000-USD 2,000 for an initial Ortho-K consultation). Consequently, it is essential to thoroughly assess the risk and cost-effectiveness before starting Ortho-K therapy for a child, especially when the clinical impact is minimal (22).

When considering any contact lens-based therapy, such as Ortho-K lenses, it is crucial to weigh potential complications. The most severe complication associated with OK lenses is microbial keratitis, which is infrequent. Other complications, like pigmented ring formation and corneal nerve pattern changes (fibrillary lines), have been presented, but these changes seem reversible (74, 80, 83, 84). Studies estimate that the probability of microbial keratitis in children using Ortho-K lenses is 13.9 per 10,000 patient-years, compared to 7.7 per 10,000 for all OK wearers (80). The predicted occurrence rate of infectious keratitis in people using daily corneal gas permeable lenses is 1.2 per 10,000. However, the rate varies between 13.3 to 19.5 per 10,000 in those wearing extended-wear soft lenses. In conclusion, microbial keratitis risk for children using overnight orthokeratology lenses is comparable to that of adults using other overnight modalities, particularly extended wear of soft contact lenses (85).

#### 3.2.2. Pharmacological intervention

Myopia has been treated with atropine eye drops, a nonselective muscarinic antagonist, for several years. A recent Cochrane review (18) established that, compared to children administered placebo eye drops, those who received atropine eye drops, pirenzepine gel, or cyclopentolate eye drops exhibited a significant deceleration in the progression of myopic refractive error over 1 year. Between the two groups, disparities of 1.00 D (95% UI 0.93–1.07) were observed, while discrepancies of 0.31 D (95% UI 0.17–0.44) and 0.34 D (95% UI 0.08–0.60) were evident among the three groups. Furthermore, the mean difference in axial elongation for the atropine group (*n* = 502) and the pirenzepine group (*n* = 326) was 0.35 mm (95% CI −0.38 to −0.31) and −0.13 mm (95% CI −0.14 to −0.12), respectively, both exhibiting lower values in comparison to the placebo group. Based on these findings, Walline and colleagues determined that antimuscarinic topical medications effectively slowed myopia progression in myopic children. Nonetheless, it is crucial to acknowledge that pirenzepine eye drops have been discontinued to mitigate myopia and are no longer accessible as a treatment alternative.

In the ATOM-1 study conducted by Chua et al. (86), the researchers found a significant decrease in the average progression of myopia among participants treated with 1% atropine (−0.28 ± 0.92 D) compared to those in the placebo group (−1.20 ± 0.92 D) over a two-year. Additionally, the 1% atropine group exhibited a stable axial length (−0.02 ± 0.35 mm), while a significant increase in axial length (0.38 ± 0.35 mm, *p* < 0.001) was observed in the placebo control group. Consequently, there was a 77% decrease in myopia progression within the 1% atropine group relative to the placebo group over 2 years. Nonetheless, the study design exhibited certain limitations, as the utilization of a high atropine concentration was linked to a noticeable rebound effect following the discontinuation of eye drops. A year after ceasing treatment, myopia progression in the study group was −1.14 ± 0.8 D/year, while it was −0.38 ± 0.39 D/year in the control group (87). In the ATOM-2 study (88), lower atropine eye drop concentrations (0.5, 0.1, and 0.01%) corresponded to myopia progression rates of −0.30 ± 0.60 D, −0.38 ± 0.60 D, and − 0.49 ± 0.63 D, along with AL measurements of 0.27 ± 0.25 mm, 0.28 ± 0.28 mm, and 0.41 ± 0.32 mm over 2 years. Utilizing reduced atropine concentrations led to a lesser increase in pupil size and a diminished impact on accommodation amplitude. After 1 year of washout, the 0.01% atropine group exhibited the least myopia development over 3 years. However, the study lacked a placebo control group, and the 0.01% atropine group had comparable axial length elongation to the previous placebo group in the ATOM1 study. Nevertheless, 0.01% atropine eye drops have become a prevalent therapeutic intervention for myopia prevention.

In response to the shortcomings of the ATOM-2 study, researchers carried out the Low-Concentration Atropine for Myopia Progression (LAMP) studies (89–91). This study evaluated three different atropine concentrations (0.05, 0.025, and 0.01%) in children with myopia. The trial outcomes showed that all three atropine concentrations effectively reduced myopia progression over 2 years. The 0.05% concentration displayed the most significant reduction (−0.74 D), and the 0.01% concentration exhibited the smallest decline (−0.46 D) (90). Additionally, LAMP studies found that low-concentration atropine eye drops were safe and well endured by the participants, with no significant side effects observed. Another meta-analysis (92) evaluated the effects of eight different concentrations of atropine on myopia progression in children and compared their safety and efficacy. The research included 3,272 people who participated in randomized controlled trials, and 30 pairwise comparisons were made. According to the findings, the atropine concentrations of 1, 0.5, and 0.05% were the most effective. In addition, it was shown that a concentration of 0.05% was the most advantageous for myopia progression. Atropine can lead to pupil dilation and hindered visual focusing, even at concentrations as low as 0.01%. Depending on the dosage, sensitivity to light and challenges with near vision might occur. Nevertheless, using photochromic lenses, multifocal lenses, or a combination of both can mitigate these effects. The research conducted in the Atropine for Myopia Treatment 2 Study (88) revealed that among children administered 0.5, 0.1, and 0.01% atropine, the proportions seeking combined photochromic progressive addition lenses were 70, 61, and 6%, respectively. The LAMP Study (89) pointed out that, among more than 400 children who were randomly assigned to receive either 0.01, 0.025%, or 0.05% atropine or a placebo, the demand for photochromic or progressive addition lenses remained consistent, regardless of the atropine dosage administered. Across all groups, including the placebo group, it was observed that roughly 30 to 40% of the children necessitated photochromic eyewear. In addition, progressive addition glasses were required by four participants, with one individual belonging to the placebo group. In these clinical trials, the most frequently encountered ocular side effect was allergic conjunctivitis, which impacted 3 to 7% of the children in each group, encompassing the placebo group in the Low-Dose Atropine for Myopia Progression Study. This suggests the cause may be attributable to the preservative or another constituent in the solution.

As a result of the probability of systemic penetration, the administration of topical atropine may result in a range of unfavorable effects, including dryness of the skin, mouth, and throat, tachycardia, restlessness, facial flushing, and irritability (93). Despite its use in numerous clinical trials, no systemic adverse effects have been reported with atropine for managing myopia (87–89) and amblyopia in children (94). The potential protective effect of myopia against age-related macular degeneration (AMD) (95) may be attributed to a longer axial length resulting in lower light flux density (96). This effect counters the risk of increased retinal light exposure and the development of AMD associated with atropine-induced mydriasis. Further research is required to address the potential concerns surrounding the risk of near vision loss in younger patients due to persistent partial cycloplegia. While topical atropine presents a minimal risk of vision loss, particularly at lower doses, higher concentrations may necessitate using photochromic glasses or soft contact lenses.

A significant proportion of children continue to exhibit poor efficacy when treated solely with lower concentrations of atropine. Prior studies have consistently shown that combined treatment yields superior efficacy to monotherapy at the 1-year follow-up (97–102). In previous studies (98, 101), combining 0.01% atropine (ATP) with defocus incorporated multiple segments (DIMS) showed significant reductions in myopia progression and axial elongation compared to DIMS alone and single vision (SV) lenses. Children receiving the combined treatment exhibited a 46% reduction in myopia progression and a 54% reduction in axial elongation over 1 year, while those treated with DIMS alone had a 21% decrease in myopia progression and a 26% reduction in axial elongation (101). Similar findings (98) were observed in a European study, where DIMS and atropine, either alone or in combination, effectively reduced myopia progression. Combining DIMS and atropine showed the most substantial reduction. These studies demonstrate the potential benefits of combining DIMS and 0.01% ATP for managing myopia in children and highlight their effectiveness in slowing myopia progression. Another combination therapy is to combine 0.01% atropine with soft multifocal contact lenses (SMCL), which was investigated in the Bifocal & Atropine in Myopia (BAM) study for controlling myopia progression and axial elongation, compared to SMCL alone. The study included three groups: Bifocal + Atropine, Bifocal, and Single Vision. Following a three-year observation period, the results revealed that the addition of 0.01% atropine to soft multifocal contact lenses (SMCL) with a + 2.50-D add power did not show superior myopia control compared to SMCL alone (99).

The effectiveness of combining ortho-k lenses and 0.01% atropine therapy in reducing axial elongation in myopic children was demonstrated in 2 years randomized trial (100). This integrated approach was found to be 28% more effective than ortho-k monotherapy. The study retrospectively indicated that the combination treatment of orthokeratology and 0.01% atropine could be a viable technique for reducing axial length elongation in myopic children. Notably, this approach appeared particularly effective in myopic children with lower degrees of myopia. Another study (102) presents the findings of a 2-year randomized controlled trial examining the efficiency of atropine, ortho-k, and combined treatment for myopia in children aged 8–12 years. Results show that all interventions significantly reduced axial elongation. Combined treatment demonstrated greater effectiveness compared to monotherapies. Age-dependent effects were observed, suggesting ortho-k may be particularly effective in younger children. These findings emphasize the potential of combined treatment and age-related variations in managing myopia in children aged 8–12.

## 4. Discussion

A one-diopter increase in myopia has been linked with a 67% increase in the prevalence of myopic maculopathy. Consequently, it is plausible that reducing myopia by merely one diopter could lead to a 40% decrease in the risk of developing myopic maculopathy (103). Furthermore, various epidemiological studies have noted differences in childhood myopia prevalence between Asian and White individuals living in the same country, implying that ethnicity could be a contributing factor (104, 105). This situation raises questions about the need for intensive public health measures to prevent myopia progression in regions with low myopia prevalence. Nevertheless, avoiding any degree of myopia can reduce individual burden. The discrepancy in myopia prevalence may be explained by the possibility of greater genetic susceptibility to myopia among Asians or a more rapid progression of the condition. As a result, myopia prevention may require different approaches depending on geographical location and individual circumstances. Generally, it is necessary to periodically review and update current options and guidelines (106).

Several behavioral prevention strategies have been proposed for myopia control, including increased engagement in outdoor activities (26, 107) and decreased near-work activities (37, 41). It is important to note that not all children will develop myopia, and predicting the final refractive error remains uncertain (108). Delaying myopia onset could slow progression, as rates tend to be age-dependent. Indication advocates that if the onset of myopia is deferred until the end of primary school, a limited number of children with delayed onset will progress to high myopia (109). Despite most epidemiological studies indicating that increased time spent outdoors may not mitigate ocular growth, the impact of seasonal variations on the progression of myopia suggests that this phenomenon may be influenced in a manner that aligns with the impact of educational demands and outdoor exposure time on myopia development (110).

Furthermore, increased smartphone exposure has also been linked with an advanced risk of myopia, and school-based intervention programs have demonstrated promise in reducing screen time and slowing myopia progression. Being outdoors generally leads to less close-up work, as children usually do not participate in such activities during outdoor playtime. In a primary school in Baoji, China, preventive measures have been implemented to discourage children from getting too close to their study materials (111). Specifically; bars have been installed above each desk to serve as a physical barrier. China has recently strengthened legislation to restrict video game screen time for minors under 18 to 3 h weekly (112).

A network meta-analysis (17) comprising 30 randomized controlled trials with 5,422 eyes investigated 16 different treatments for controlling myopia. Results indicated that exposure to high, moderate, or low doses of atropine significantly delayed the onset of myopia when compared to SVS lenses or placebo. Additionally, orthokeratology and peripheral defocus modifying contact lenses showed significant effects, while progressive addition glasses showed minimal effects. The network analysis findings suggest that several interventions have the potential to considerably decelerate the progression of myopia when compared to single-vision spectacle lenses or placebo. Pharmaceutical therapies such as muscarinic antagonists like atropine proved the most successful. Special contact lenses, including those utilized in orthokeratology and those designed to correct peripheral defocus, exhibited substantial effects, whereas specially designed spectacle lenses had minimal impact. However, it should be noted that the higher the concentration of atropine, the greater the risk of unwanted side effects. Premyopes and younger children of 8–10 years can also benefit from the combined treatment of atropine and orthokeratology lenses (100, 102). As no direct comparisons have been made between the different treatment modalities, it is impossible to establish a specific treatment order, such as designating one as the first-choice or second-choice therapy (113).

To offer informed guidance on selecting new treatments for individuals, relying on meticulously conducted controlled studies over an extended duration is crucial. Considering the potential prolonged consequences of a treatment applied to numerous children and adolescents, it is possible that adverse effects, such as those associated with atropine, may take several decades to become apparent. Given that myopic children require some form of optical intervention, non-invasive optical methods such as wearing glasses or contact lenses do not constitute an additional procedure like administering atropine. When comparing the effects of different treatment modalities, expressing the results as a percentage of percentage may create the perception of a larger impact than what really occurred. It is also essential to consider the limited accessibility of commercial atropine eye drop medicine in different countries when considering the pharmacological approach of using low-concentration atropine eye drops. Availability can vary between regions, which should be considered when considering atropine as a treatment option for myopia. The scope of the review is limited to strategies for school children, potentially excluding relevant information on myopia prevention strategies for other age groups or populations. The study selection process may also introduce bias as it relies on the author’s interpretation of the available literature. Narrative reviews have inherent limitations, including subjectivity, bias, lack of systematic methodology leading to potential incompleteness and selective inclusion, and absence of quality assessment. Language barriers may further affect the assessment, as relevant studies published in non-English languages may not have been accessed. Lastly, the quality of the included studies may be a limitation, as some may have limitations in study design or sample size, potentially affecting the validity of the results.

## 5. Conclusion

In conclusion, the existing evidence consistently supports the effectiveness of atropine eye drops in preventing the development of myopia. However, further research is needed to determine the optimal concentration of atropine and the potential benefits of combining atropine eye drops with other optical devices. Other interventions like orthokeratology, soft contact lenses with myopia control features, and spectacle lenses with aspherical lenslets have also shown promising results. However, despite their effectiveness, the widespread adoption of these treatments in clinical practice may be hindered by side effects, cost, complexity, and limited efficacy. Staying updated on myopia research helps clinicians and patients choose the best strategies to manage myopia and promote optimal eye health in children.

## Author contributions

FT and HG designed the study. FT wrote the original draft. RM, XW, XL, QB, and JL improved the manuscript. All authors contributed to the article and approved the submitted version.

## Funding

This work was financially supported by the National Natural Science Foundation of China (82070923), the Taishan Scholars Program (201812150), and Academic Promotion Program of Shandong First Medical University (2019RC009), and Youth Science Foundation of Shandong First Medical University: (G0402-202202-036).

## Conflict of interest

The authors declare that the research was conducted in the absence of any commercial or financial relationships that could be construed as a potential conflict of interest.

## Publisher’s note

All claims expressed in this article are solely those of the authors and do not necessarily represent those of their affiliated organizations, or those of the publisher, the editors and the reviewers. Any product that may be evaluated in this article, or claim that may be made by its manufacturer, is not guaranteed or endorsed by the publisher.
